# Advances in combined neuroendocrine carcinoma of lung cancer

**DOI:** 10.3389/pore.2024.1611693

**Published:** 2024-05-14

**Authors:** Zesen Han, Fujun Yang, Fang Wang, Huayu Zheng, Xiujian Chen, Hongyu Meng, Fenglei Li

**Affiliations:** ^1^ Hua Country People’s Hospital, Anyang, Henan, China; ^2^ Department of Medical Oncology, Sanmenxia Central Hospital, Henan University of Science and Technology, Sanmenxia, China

**Keywords:** small cell neuroendocrine carcinoma, large cell neuroendocrine carcinoma, adenocarcinoma, squamous cell carcinoma, lung cancer

## Abstract

Lung cancer incidence and mortality rates are increasing worldwide, posing a significant public health challenge and an immense burden to affected families. Lung cancer encompasses distinct subtypes, namely, non-small-cell lung cancer (NSCLC) and small-cell lung cancer (SCLC). In clinical investigations, researchers have observed that neuroendocrine tumors can be classified into four types: typical carcinoid, atypical carcinoid, small-cell carcinoma, and large-cell neuroendocrine carcinoma based on their unique features. However, there exist combined forms of neuroendocrine cancer. This study focuses specifically on combined pulmonary carcinomas with a neuroendocrine component. In this comprehensive review article, the authors provide an overview of combined lung cancers and present two pathological images to visually depict these distinctive subtypes.

## Introduction

Globally, the morbidity and mortality rates of lung cancer are increasing [1], making it a significant public health concern and burden for families [2]. Approximately 75% of the 2.20 million newly diagnosed lung cancer patients will succumb to the disease within 5 years [3–5]. Lung cancer comprises distinct subtypes, such as non-small-cell lung cancer (NSCLC) and small-cell lung cancer (SCLC). The mortality rate of NSCLC has been shown to improve from 2013 to 2016 following diagnosis [6], due to advances in screening, early patient management, immunotherapy, and other interventions [7]. However, SCLC remains challenging due to its propensity for relapse and higher mortality rates accounting for up to 15% of all lung cancers [8]. Despite decades of research focused on targeted treatments based on biomarker selection and immunotherapy, SCLC continues to be one of the most difficult-to-treat tumorigenic diseases [9].

In 2021, the WHO classified the lung tumors since 2015 [10] into five categories: 1) Small cell carcinoma, 2) Large cell neuroendocrine carcinoma, 3) Adenosquamous carcinoma (if both components ≥10%), 4) Adenocarcinoma, squamous cell carcinoma, adenosquamous carcinoma, or large cell carcinoma with unclear immunohistochemical features, 5) Pleomorphic, spindle cell, and/or giant cell carcinoma for the resection specimens. However, in clinical work, researchers have observed an additional subtype. In pathology studies, neuroendocrine tumors can be further divided into four types: typical carcinoid tumor, atypical carcinoid tumor, small cell carcinoma, and large cell neuroendocrine carcinoma based on their combined features [11]. This study focuses specifically on combined pulmonary carcinoma with a neuroendocrine component.

### Combined small cell neuroendocrine carcinoma with adenocarcinoma

The current consensus is that small-cell lung cancer is transferred from adenocarcinoma following treatment with EGFR tyrosine kinase inhibitors [12]. This phenomenon was initially reported in a female patient diagnosed with adenocarcinoma who received erlotinib in 2007. After prolonged treatment, a biopsy of the same site revealed SCLC based on the exon 19 mutation of the epidermal growth factor receptor (EGFR), which was consistent with primary NSCLC [13]. Another case involved a 38-year-old patient with EGFR exon 21 L8585R lung adenocarcinoma who developed SCLC transformation after receiving regular erlotinib treatment for 18 months [14]. Two hypotheses have been proposed to elucidate the pathogenesis of SCLC transformation [15]. The majority of researchers posit that this transformation arises from resistance to tyrosine kinase inhibitors (TKIs) [16] targeting EGFR, ALK, and ROS1, or immunotherapies [17]. Five resistant tumors were found to harbor mechanisms such as the EGFR T790M mutation, MET gene amplification, EGFR amplification, mutations in the PIK3CA gene and others associated with the epithelial-to-mesenchymal transition. These transformed tumors from NSCLC to SCLC showed sensitivity to standard SCLC treatments. However, due to significant heterogeneity in resistance mechanisms and different prognoses among cases [18, 19], individualized therapeutic strategies are required. The transformation can be detected by mutations in biomarkers such as EGFR, tumor protein p53 (TP53), RB transcriptional corepressor 1 (RB1), and SRY-box transcription factor 2 (SOX2) before and after transformation [20].

However, it should be noted that this transformation may also be pseudo. In a study conducted by Rui Li et al. in 2021 involving 11 cases previously diagnosed as SCLC, only one sample did not exhibit any SCLC elements within the primary adenocarcinoma sections. This case was defined as true small-cell transformation (SCT) [21]. In other words, there were instances where SCLC components coexisted within the adenocarcinoma but were considered pseudo-SCT. The observation of RB1 deletion and mutant TP53 overexpression in either pseudo-SCT or true SCT cannot exclude the possibility of combined SCLC with adenocarcinoma.

Meanwhile, 34 cases of combined high-grade neuroendocrine carcinoma (HGNEC) were reported with 48% of subjects with combined HGNEC and adenocarcinoma having a lepidic adenocarcinoma component suggesting that HGNEC can develop in association with pre-existing adenocarcinoma which is often retained [22]. In the same year, a case based on combined SCLC with non-small cell carcinoma component was reported, in which two distinct neoplastic components were found. One consisted of small-sized cells without giant cell carcinoma shown in the biopsy specimen while the other was verified as combined SCLC with a giant cell carcinoma through histopathological examination of the lobectomy specimen [23]. These findings indicated that SCLC and adenocarcinoma can coexist in the same patients. The results cannot be solely attributed to the transformation from adenocarcinoma, and it is imperative not to overlook the significance of combined SCLC.

The origin of two different tumor components may be from the same pluripotent epithelial precursor cell due to loss of heterozygosity (LOH) in the different tumor areas [24]. Additionally, IL-16 rs859 was found to have a statistically significant susceptibility to lung small-cell carcinoma and adenocarcinoma [25].

Expression of stem cell transcription factors (scTF) has been detected in both small cell carcinoma and non-adenocarcinoma in prostate cancer, influencing the transformation from adenocarcinoma [26]. Inhibition of exportin 1 has been suggested as a potential therapeutic target for the prevention or treatment of neuroendocrine transformation of lung and prostate adenocarcinomas [27]. Nowadays, a platinum plus etoposide chemotherapy regimen is preferred to treat patients with SCLC transformation based on EGFR mutation. However, new strategies, such as immune checkpoint inhibitors are being explored [28]. The DLL3-directed antibody-drug conjugate rovalpituzumab tesirine [29] and its application have been considered for the unique EGFR mutant SCLC transformation cancer [30]. Serum neuron-specific enolase (NSE) may serve as a novel marker for predicting neuroendocrine tumor transformation [31].

### Combined large cell neuroendocrine carcinoma with adenocarcinoma

The incidence of large cell neuroendocrine carcinoma (LCNC) in lung cancer is only 3% [32], and it has a poor prognosis due to its rarity, aggressiveness, and distinct treatment approach [33]. In 2009, E Cakir et al. identified different combinations of histological subtypes in lung cancer, such as adenosquamous carcinoma, combined neuroendocrine tumors, and biphasic tumors. Combined neuroendocrine carcinoma consists of SCLC + nonneuroendocrine carcinoma (NNEC), SCLC + LCNC, and LCNC + NNEC, it has been revealed that patients with combined neuroendocrine tumors had more advanced stages and vascular invasion compared to those with single histology types, their 2-year survival rate was only 25% [34]. Moreover, accurate differentiation of LCNC from atypical carcinoids is challenging with the limited tissue samples obtained through lung biopsy [35].

These findings highlight the existence of evidence of combined large-cell neuroendocrine carcinoma. The diagnosis of LCNC relies on histopathological examination while immunohistochemical (IHC) features provide precise and accurate identification. Neuroendocrine components strongly express markers such as Chromogranin A (CgA), Synaptophysin (Syn), and neural cell adhesion molecule 56 (CD56). On the other hand, thyroid transcription factor 1 (TTF-1) is associated with EGFR mutations, while NapsinA is highly specific for lung adenocarcinoma [36]. P40 serves as an excellent marker for distinguishing between squamous cell carcinoma and lung adenocarcinoma [37], similar to P63. Among combined LCNC cases, adenocarcinoma is the most common combination, accounting for approximately 70% of cases [38]. A retrospective study of surgical resection of combined LCNC included 96 patients, 71 of whom were diagnosed as having LCNC combined with adenocarcinoma.

**FIGURE 1:**
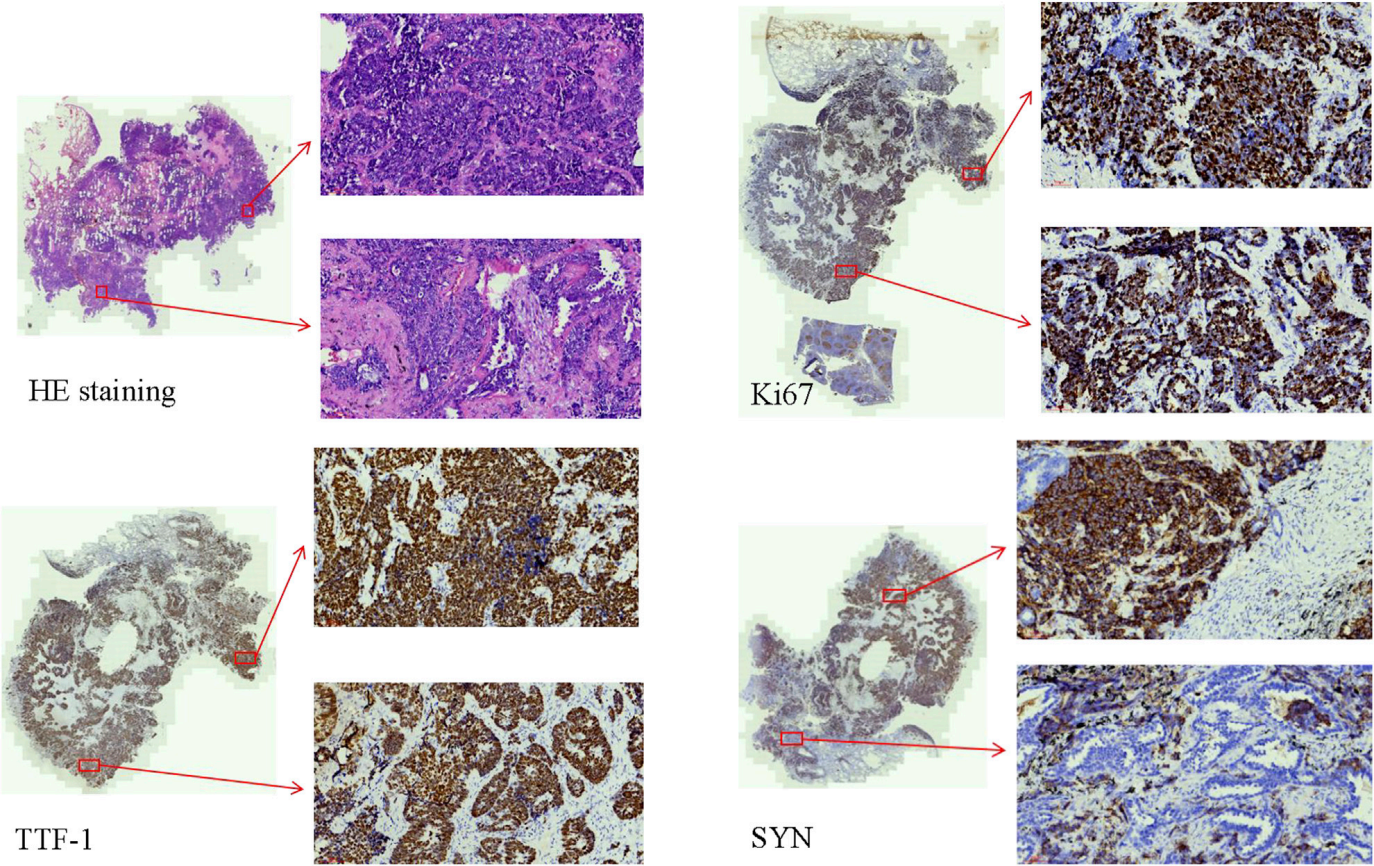
The HE staining and IHC of the combined LCNC with adenocarcinoma (×20). The Ki67 index was approximately 85%. TTF-1 and SYN were positive.

During clinical work, the authors encountered a case of LCNC combined with adenocarcinoma that could be diagnosed by IHC. This is a resection specimen. In this particular case, positive markers for TTF-1, SYN, CD56, CK, and P63 were observed, along with partial positivity for NapsinA. However, CgA and P40 showed negative results. The Ki67 index was approximately 85% (Figure 1). Metastasis was observed in 22 of 33 lymphatic nodes.

Adjuvant chemotherapy, particularly etoposide-based chemotherapy, proved to be a beneficial option [39]. Furthermore, it is crucial to confirm the importance of adjuvant chemotherapy (especially using the small cell carcinoma regimen) to improve patients’ outcomes [40]. Nevertheless, immunotherapy rarely provides benefits when combined with LCNC treatment. Further research should be done [41].

### Combined large cell neuroendocrine carcinoma with squamous cell carcinoma

Squamous cell carcinoma is a subtype of non-small cell carcinoma, which also constitutes the composition in combined LCNC. A case of LCNC of the lung with carcinoid syndrome was reported involving a 76-year-old woman who underwent computed tomography that revealed a liver mass originating from the lung as diagnosed by biopsy. The pathology analysis of the lung biopsy demonstrated combined LCNC and squamous cell carcinoma. The tumor area tested negative for TTF-1 but positive for cytokeratin14 (CK14) and P40 [42]. Despite receiving chemotherapy following the diagnosis, the patient died 50 days after hospital admission due to her deteriorating physical condition. In 2004, another case was reported in which a patient diagnosed with combined LCNC as part of squamous cell carcinoma [pT4 (pm) N2M0] on postoperative histological tissue remained in good health for 9 months until the article was published [43]. In addition, other similar cases have been documented [44].

**FIGURE 2:**
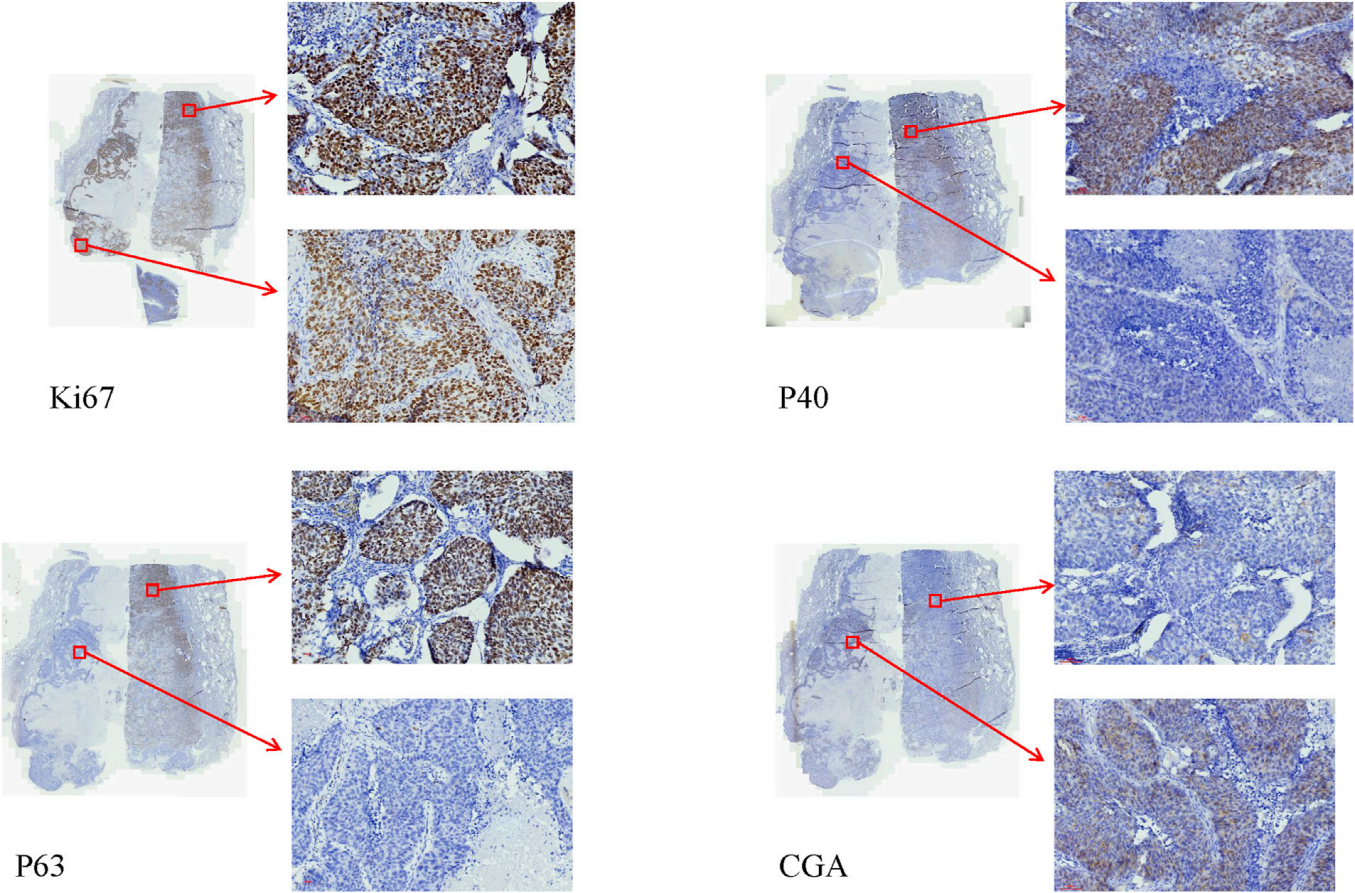
The IHC of the combined LCNC with squamous cell carcinoma (×20). The Ki67 index was approximately 80%. The sections of the P40, and P63 of squamous cell carcinoma were positive. The sections of the P40, and P63 of LCNC were negative. The sections of CgA of LCNC were positive. The sections of CgA of the squamous cell carcinoma were negative.

The authors encountered a case of combined LCNC with squamous cell carcinoma that was diagnosed using IHC and pathological features. In this particular case, the IHC markers: CK, and CK17 were found to be positive. Additionally, the tumor sections were positive for CgA, P40, and P63. However, the markers CK7, TTF-1, NapsinA, SYN, CD56, and CD117 were negative. The Ki67 index was approximately 80%. Notably, organoid and palisading patterns were observed in the majority of the lung tumor cells within the tumor areas. Furthermore, another section revealed a prominent presence of atypical cells exhibiting keratinization (Figure 2). Importantly, no lymphatic node metastasis was detected.

According to the IHC, the patient was diagnosed with combined LCNC and squamous cell carcinoma due to the presence of two distinct elements. Additionally, molecular testing could be used to satisfy the criteria of precision medicine [45].

Conversely, miR-31 is found to be upregulated in adenocarcinoma, squamous cell carcinoma, and large-cell neuroendocrine carcinoma of the lung, while it is not overexpressed in small-cell carcinoma or carcinoids. MiR-31 has been identified as a potential therapeutic target that promotes tumor growth in mice of xenografted human adenocarcinoma and squamous cell carcinoma cell lines but not in large- or small-cell carcinoma lines [46]. The Ki-67 proliferation index cutoff of 55% could predict the prognosis of LCNC and combined LCNC, with combined LCNC patients having longer overall survival (OS) when diagnosed with adenocarcinoma compared to those diagnosed with squamous cell carcinoma [47].

Notably, there was a case report demonstrating the coexistence of LCNC with adenocarcinoma, and squamous cell carcinoma. Hematoxylin—Eosin staining revealed that the tumor consisted of 40% acinar adenocarcinoma, 10% mucous adenocarcinoma, 40% LCNC, and 10% poorly differentiated squamous cell carcinoma. The markers TTF-1, SYN, and P40 exhibited positive expression in the correlative tumor [48]. The researcher indicated that surgical resection along with adjuvant chemotherapy using SCLC regimen may improve Disease Free Survival (DFS) and OS.

Treatment of combined LCNC with squamous cell carcinoma could include immune checkpoint inhibitors after multimodality therapy incorporating cytotoxic anticancer drugs and radiotherapy. A 60-year-old man diagnosed with LCNEC combined with squamous cell carcinoma and staged as T2aN0M0 stage IB through histopathology showed a favorable response to treatment and achieved a survival period exceeding 5 years [49].

The identical phenomenon of merging two or more compounds has also been observed in other parts of the body, such as the head and neck region [50] and the uterine cervix [51].

In this comprehensive review, the authors meticulously summarize the various types of combined lung cancers, including the concurrence of combined small cell neuroendocrine carcinoma with adenocarcinoma, combined LCNC with adenocarcinoma, and combined LCNC with squamous cell carcinoma.

Interestingly, there seems to be no literature available on the coexistence of small-cell neuroendocrine carcinoma and squamous cell carcinoma in the lung. This particular type of lung cancer has a significantly poorer prognosis, and thus, research concerning its treatment is currently underway. The review also highlights the importance of understanding the different combinations of lung cancer subtypes, as this knowledge can greatly contribute to more tailored and effective treatment strategies.
